# Barriers and facilitators of nutrition assessment, counseling, and support for tuberculosis patients: a qualitative study

**DOI:** 10.1186/s40795-021-00463-x

**Published:** 2021-10-13

**Authors:** Meaza Girma Degefa, Afework Mulugeta Bezabih, Znabu Hadush Kahsay, Abate Bekele Belachew

**Affiliations:** 1 Department of Public Health, College of Medicine and Health Sciences, Wachemo University, Hossana, Ethiopia; 2 Department of Nutrition and Dietetics, School of Public Health, College of Health Sciences, Mekelle University, Mekelle, Ethiopia; 3 Department of Environmental Health and Behavioral Sciences, School of Public Health, College of Health Sciences, Mekelle University, Mekelle, Ethiopia; 4 School of Public Health, College of Medicine and Health Sciences, Arba Minch University, Arba Minch, Ethiopia; 5 Center for Environmental and Respiratory Health Research, Faculty of Medicine, University of Oulu, Oulu, Finland; 6 Biocenter Oulu, University of Oulu, Oulu, Finland

**Keywords:** Nutrition assessment, Nutritional support, Tuberculosis, Ethiopia

## Abstract

**Background:**

Nutrition has a substantial role in the prevention, treatment, and cure of tuberculosis. Thus, nutrition assessment, counseling, and support (NACS) have been implemented as part of tuberculosis treatment. However, evidence on the barriers and facilitators (enablers) of its implementation is lacking.

**Objective:**

To explore barriers and facilitators of implementation of NACS for tuberculosis patients.

**Methods:**

An exploratory qualitative study was conducted in public health facilities and health offices of Mekelle City, Northern Ethiopia. We conducted 17 interviews using purposively selected key informants comprising health professionals (*n* = 12) and tuberculosis patients (*n* = 5). Interviews were tape-recorded, transcribed verbatim, and coded and analyzed using a thematic approach in ATLAS.ti 7 software.

**Results:**

Barriers were identified at three levels -organization, care provider, and patient levels. Suboptimal nutritional supply, lack of supportive supervision, lack of adequate workforce, staff turn-over, the sudden withdrawal of partners, and weak link with social service were the barriers at the organization level. Lack of commitment was reported as the only barrier at the care provider level, and socioeconomic status of patients, sharing and selling of supplies, perceived improved status, and perceived stigma were identified as the major barriers for the implementation of nutrition assessment, counseling, and support service. While training, availability of measurement and educational tools, the inclusion of nutrition indicators in the tuberculosis register, and the presence of collaborating partners were identified as facilitators at the organizational level. Patients’ motivation to know their health status was reported to be a facilitator at the patient level.

**Conclusions:**

Organization, care provider, and patient-level barriers and facilitators were found to influence the implementation of NACS. Hence, multilevel factors should be considered to successfully implement the program and to gain its potential impact.

**Supplementary Information:**

The online version contains supplementary material available at 10.1186/s40795-021-00463-x.

## Background

Tuberculosis (TB) is an infectious disease that remains a leading cause of morbidity and mortality, with 10 million new TB cases and 1.3 million TB deaths worldwide in 2017 [1]. The highest disease burden occurs in developing countries where undernutrition and poverty are major problems [2]. Ethiopia, with 172,000 new TB cases, 5500 multidrug-resistant TB (MDR-TB), and 25,000 deaths in 2017, is still one of the high TB burden countries which could be explained by an unacceptably high prevalence of malnutrition [1]. Even though the burden of TB is extensive, TB treatment has averted 54 million deaths worldwide between 2000 and 2017 [1].

Nutrition and tuberculosis have a bidirectional relationship by which both are risk factors for each other [3]. Undernutrition is the main risk factor for the development of TB by severely affecting cell-mediated immunity which is the principal host defense against TB [3]. The risk of progression from infection to disease increases substantially in undernourished individuals. Severe undernutrition at diagnosis has been reported to be associated with a higher risk of death [4–6]. It also increases the risk of TB drug toxicity and relapse [7].

Decreased appetite, nutrient malabsorption, and altered metabolism which is caused by TB itself can adversely affect nutritional status leading to both micronutrient [8, 9] and macronutrient deficiency [10–12]. Undernutrition is reported to be high among TB patients [10–13] and is also known to result in poor TB treatment outcomes [6, 9, 14]. In Ethiopia, evidence indicates that 39.7–53% of TB patients are undernourished [10, 12].

Nutrition and proper diet are essential for the well-being and health of all people including TB patients [3]. Effective nutrition-specific and nutrition-sensitive interventions have been implemented to improve the nutritional status of vulnerable groups particularly children and women [15]. Though evidence about the potential interventions to improve the nutritional status of TB patients is limited, a pre-post intervention study reported that nutrition education can improve the nutritional status of TB patients [16]. More importantly, given the complex relationship between nutrition and infections [17], nutrition assessment, counseling, and support (NACS) is suggested for patients with HIV and/or TB. NACS is a client-centered program for integrating nutrition care into routine clinical services. This approach has become an important strategy to reduce the severity of the illnesses and improve treatment outcomes and quality of life for patients with HIV and/or TB [18–20]. The World Health Organization (WHO) [19] and national [21] guidelines recommend periodic nutritional assessment, counseling on diet, and nutritional support that can help TB patients to maintain or increase their food intake and adhere to TB medications [13, 22].

According to the Ethiopian TB guideline, assessment of the nutritional status of every TB patient using anthropometric indices is undertaken by health care providers at initial assessment and preparation for TB treatment, at the end of the intensive phase of TB treatment, and upon documenting the unintentional loss of weight during TB treatment. Accordingly, nutritional support will be provided for all patients with TB depending on the degree of malnutrition and the age of the patient. Nutritional counseling (for example, counseling to eat more and a variety of foodstuffs important to achieve a healthy balanced diet) is provided for all patients with active TB as part of routine adherence upport to TB [21, 23].

In Ethiopia, a country with a high burden of TB and Malnutrition, control and prevention of TB is among the priority health programs as indicated in Health Sector Transformation Plan (HSTP) and also working towards END TB strategy by adopting the new Global TB Strategy [21, 24]. In order to achieve this goal, given the significant role of nutrition and proper diet to TB patients, NACS have been indicated as one of the TB treatment strategies [19, 21]. Hence, since 2016, the national nutrition program II initiative has been taken to address the nutritional requirements of individuals with infections, specifically tuberculosis. Despite the recognition of NACS as routine TB care, information on the barriers and facilitators (enablers) of NACS implementation in the Ethiopian context is lacking. Food and Nutrition Technical Assistance (FANTA) activity reports on the implementation of NACS for HIV patients in Ethiopia showed a lack of integrated HIV and nutrition training, inconsistent provision of training for health professionals, and weak supervision of NACS service have known to challenge the implementation of NACS [25]. These challenges have led health care providers to focus primarily on HIV services and to assume not accountable for providing NACS service. The nutrition service for HIV patients is reported to be heavily dependent on collaborating partners that has hindered the effective implementation of the NACS [25]. Supportive management and provision of nutrition supply are reported to motivate the healthcare providers to improve the quality of nutrition services in Uganda [26]. Integration of data recording tool that includes nutrition parameters in the existing health service registers is suggested to improve the implementation of NACS in Zambia [27]. Scholars suggested that effective implementation of evidence can be affected by factors that operate at various levels; the patient, care providers, and organizational level [28].

Therefore, this study aimed to investigate the barriers and facilitators (enablers) of implementation of NACS from the organization, care providers’, and patients’ perspectives using an exploratory qualitative study.

## Methods

### Study setting

This study was conducted in public health facilities and health offices of Mekelle City, Tigray regional state, Northern Ethiopia in April 2019. The City is comprised of seven sub-cities/districts that have 10 health centers and 4 hospitals of which one is a tertiary hospital, and the rest are general hospitals. TB treatment and care is one of the services provided at all health facilities. Early diagnosis of tuberculosis, systematic screening of contacts, high-risk groups, and treatment of all people with TB including drug-resistant TB are of highest priority services (Regional Health Bureau annual profile, 2018, unpublished). The NACS for the patients is provided according to the national guideline that integrated nutrition as the main component of TB treatment and care [23]. All the care providers of the TB program or care at various levels of the health system including regional and woreda (district) health offices and health facilities are trained on recent TB treatment protocol [23]. According to the regional government report of 2018, the TB retreatment cases were 61(8%) with a treatment success rate of 94% for both bacteriological and clinically confirmed TB case. In addition, unfavorable treatment outcomes such as death, loss to follow-up, treatment failure and moved to MDR were 5, 1, 0.8, and 0.3%, respectively (Regional Health Bureau annual profile, 2018, unpublished).

### Study design and participants

An exploratory qualitative study was employed to generate in-depth information on the barriers and facilitators of NACS implementation. We approached 17 purposively selected key informants. Participants consisted of health professionals (nurses and public health officers) such as four TB program coordinators at woreda (district) and regional health office, two health facility managers, six care providers (TB focal persons), and five TB patients from various health facilities. They were identified as key informants based on their exposure to TB care and support as a manager, care provider, or patient. Facility managers, TB program coordinators, and care providers were recruited based on the duration of stay in the position (at least 6 months of experience) related to TB care and support. While adult TB patients (greater or equals to 18 years) who have received at least 2 months of TB treatment and differ by the type of TB (Drug susceptible-TB (DS-TB), MDR, and TB/HIV), type of facility (health center and hospital) they are receiving care were recruited to include patients with different disease type and possible treatment category.

### Data collection procedures

Interviews were conducted using a semi-structured interview guide, adapted from Grol’s model for barriers and incentives of change in health care [28]. Based on this framework, barriers and facilitators of the implementation of NACS could operate at various levels including the individual patient, care providers, and organization context [28]. The guide was further enriched based on the WHO and a national TB treatment guideline [19, 21]. The guide comprised open-ended questions with certain main topics to be covered such as routine TB service, routine NACS services, and the barriers and facilitators for implementation of NACS at the patient, care provider (professional), and organization aspects (Additional file 1). It included a total of six questions related to background information, 17 main questions with at least one to six follow-up questions. The interviews were conducted by researchers from the School of Public Health, Mekelle University (including authors MGD, female, and ABB, male, and one male colleague). The interviewers and interviewees did not know each other before the start of the study. The interviews were conducted on a face-to-face basis between the data collector and a participant using the local language [Tigrigna or Amharic]. The interviews were tape-recorded, and notes were taken for a non-verbal explanation on the topic of discussion. Before actual data collection, the participants were asked permission, choose a convenient time and place for the interview to reduce the possibility of interrupting the interview for office or other personal commitments. Each interview took an average of 54 min (range: 30 to 88 min) and was estimated to be 60 min before the study. The interviews were done in a private room to ensure the privacy and quality of recordings.

### Data quality control

The data collection guide was piloted using three study participants from the study setting. The information was then transcribed, translated, and analyzed before the actual study hence emerging insights were incorporated into the interview guide. Then, a discussion was made by the researchers on the final version of the interview guide, the techniques of interview and questioning, participant selection, and ethical issues. Accordingly, the interviewers were well informed to withhold their prior experiences and avoid leading questions to minimize perception bias during data collection and transcription. Participants were probed using follow-up questions for points that need clarification and depth. Investigators conducted daily debriefing sessions to enhance the trustworthiness of the data. We conducted data coding and identified new dimensions about the topic of the interview and information saturation daily [29]. Saturation was determined when similar ideas and insights were repeatedly heard, considering the aim of the study. We completed the COREQ 32-item checklist to inform clear and comprehensive reporting of qualitative studies (Additional file 2).

### Data analysis

Our analysis was based on all seventeen interviews. The recordings were transcribed and translated into English. Additional information was not sought from the interviewees afterward. The translated transcriptions were imported to ATLAS.ti version 7 software for coding and analysis. An initial coding scheme was done by the authors MGD and ZHK after reading the transcripts. The final coding and analysis were done by the principal investigator, MDG. Open coding was employed to break down, conceptualize, and put back the data. Then, similar codes were systematically organized within non-repetitive themes using a thematic analysis approach as suggested by Braun and Clark [30]. Handwritten notes which are additional information for the data were also reviewed. During the analysis, memos were recorded to capture the ideas and reflections of the participants. The emerged themes were finally organized into three levels namely organization, care provider, and patient.

### Operational definition

#### Barriers and facilitators

Factors are considered as barriers if they hinder the implementation of NACS, while they are considered facilitators if their presence promotes or enables the implementation of NACS.

#### Health professionals

Any health personnel who are involved in the provision of TB care at different levels and positions in the health system such as health facility, district health office, and regional health office.

#### Care providers

In this study, care providers are health professionals who deliver TB care in health facilities.

## Results

### Characteristics of study participants

The educational status of the participants ranged from primary school to master’s degree (Table 1).

**Table 1:** Characteristics of study participants

	Category	Educational status	Duration of exposure to TB care ^a^
P1	MDR-TB patient	Secondary education	7
P2	TB patient	Secondary education	3
P3	TB patient	Bachelor’s degree	2
P4	TB/HIV patient	Primary education	3
P5	Retreatment TB/HIV patient	Secondary education	20
P6	Care provider	Diploma	3
P7	Care provider	Bachelor’s degree	4
P8	Care provider	Diploma	10
P9	Care provider	Bachelor’s degree	3
P10	Care provider	Master’s degree	2
P11	Care provider	Bachelor’s degree	8
P12	Facility manager	Bachelor’s degree	2
P13	Facility manager	Bachelor’s degree	3
P14	Program coordinator	Diploma	1
P15	Program coordinator	Bachelor’s degree	2
P16	Program coordinator	Master’s degree	3
P17	Program coordinator	Bachelor’s degree	4

*P* Participant, *TB* Tuberculosis, *MDR-TB* Multidrug Resistant-Tuberculosis, *TB/HIV* Tuberculosis/Human immunodeficiency virus; ^a^ It is duration of treatment (in month) for patients and duration of experience in the position (in year) for managers, coordinators, and care providers.

### Emerged themes

Emerged themes were organized to organizational, care providers, and patient-level barriers and facilitators for implementation of NACS (Table 2).

**Table 2:** List of themes for barriers and facilitators of NACS implementation

Levels	Themes	
	Barriers	Facilitators
Organization	• Suboptimal nutritional supply • Lack of supportive supervision • Lack of enough workforce • Staff turn-over • Sudden withdrawal of partners • The weak link with social service	• Training • Availability of measurement and education tools • Inclusion of nutrition indicators in the TB register • Collaborating partners
Care providers	• Lack of commitment	
Patients	• Socioeconomic status of patients • Sharing and selling of supply • Perceived improved status • Perceived stigma	• Motivation to know their status

### Organizational level barriers of NACS implementation

#### Suboptimal nutritional supply

The absence of therapeutic and supplementary foods for NACS, interruption, and the delay were evident. Participants described that due to the absence of therapeutic and supplementary foods, drug-susceptible TB patients with severe and moderate acute malnutrition are not being provided with nutrition support: *“The supply had been reduced gradually. Then, the service was forced to be interrupted through an official letter from the region that gives the direction to provide nutrition support for only HIV patients due to a shortage of nutrition supplements. So, those facilities with antiretroviral therapy (ART) service are also started to address only HIV patients” (P17).* Participants also described that interruption and shortage of nutrition supply hinder proper management and follow-up of severe and moderate acute malnutrition patients: ‘ *… it interrupts very much... We share the support that comes for HIV patients. Recently there was a supply that came for TB patients, but it is very few. It is not enough; it may get one or two patients. So, they didn’t get continuously’ (P10).* Inadequate and late financial support for MDR-TB patients reported to hinder proper management and follow-up of severe and moderate acute malnutrition patients: *“The Global Fund is still providing support for food items provision for MDR patients though it is not adequate. It is 650 birr per month per individual, but when we think what can be bought with this money, it is very difficult” (P17) and “Sometimes the money to purchase the supply comes late from the center. After it reaches the finance, the process there itself could be a barrier, because the materials are bought after tender” (P16).* However, the program coordinator explained the reason for late and inadequate support by donors is due to lack of proper financial documentation and timely report from lower to a higher level of the health system management: “*… the provided financial support has to be utilized and reported timely (liquidation has to be done timely) till to higher levels. Currently, the money given is small and it is also not utilized and reported on time. The funders believe as the money is utilized if and only if the financial report reaches them timely. Otherwise, the donors think as there is financial support provided previously and in the following round, they only provide a small amount of money. This is also a great challenge” (P17).*

Consequently, the unavailability of nutritional supply (plumpy sup and plumpy nut) in turn discouraged the TB focal persons to conduct the nutritional assessment. This was described by care providers as: “*There is no available nutritional service in our health center for TB patients. Without giving our clients any access to nutrition, how can we measure their nutritional outcomes? It is non-sense” (P6)*. And *“If there is no therapeutic food, you may not be interested to measure height and BMI as well. If you fail to take these measurements, you will not conduct other nutrition assessments. Therefore, the absence of support leads you to be reluctant to provide the whole nutrition assessment service” (P10).* This is also further strengthened by the program coordinator: “*Access to nutritional supplements at facility level is essential to assess and identify nutritional problems in TB patients. Otherwise, assessment and identification of nutritional problems are demotivating” (P15).*

#### Lack of supportive supervision

Lack of supportive supervision and follow-up decreased the care providers’ accountability and attention to the service as the TB focal persons are providing the service as per their understanding and conscience. A TB focal person explained this as: *“There is no accountability. Therefore, whether you work or not, it is for your conscience. The higher-level leaders have no know-how about what we are providing regarding assessment. What they know is as we give plumpy nut. This is a barrier because to work it with attention, accountability is very important in addition to your conscience. But there is no supervision from facility managers and higher-level supervisors. Therefore, we are doing what we believe is right” (P10).*

The care providers also reported that sustained improper implementation of nutrition support due to lack of supportive supervision as impairing the implementation: “*It makes you sad sometimes. I mean when you are doing meaningless work if there is no one who monitors this. … integration of the service has no meaning” (P10).* In addition, lack of monitoring and follow up, which was assumed helpful to correct care providers’ weakness if any, and to apprize good performance was noted to decrease the staff motivation. A TB focal person elaborated this as: “*There are no barriers other than lack of encouragements for professionals. Due to lack of strict follow-up and monitoring to this service, we provide it haphazardly” (P10).*

#### Lack of adequate workforce

Inadequate workforce and provider’s high work burden at health facilities were reported as a challenge to NACS. Participants underlined that it is difficult to implement NACS in the case of high patient flow and a limited number of care providers. The care provider would not have time to give the service for all the clients according to the recommendation. A care provider illustrated this idea as: *“Sometimes when there is only one who provides the service, it might be difficult to conduct the whole assessment related services. But when we (two professionals) are providing service it is easy for us to conduct the assessment. When there is high patient flow, it is difficult to give attention to the whole service and provide it as needed” (P10).* A program coordinator further stated this idea as: “*There is only one TB focal person in each facility but if the number [of health care providers] is increased, it would have been easier to assess the nutritional status and counsel” [P15].* Participants also explained assigning only one focal person with additional workload other than the provision of service for TB clients to be a barrier to provide all the nutrition services for the TB clients: “*First, it is the patient load, for example, a professional can have limited time to provide all the services. The human resource shortage is one challenge. Some of them have another additional duty” (P15).* This idea was further explained by a patient as: *“The care provider is too busy. She is alone to provide us the services. Ideally, she is assigned to us (TB patients), but there are also other patients who seek her services. So, it is difficult to cover the services for all TB patients and other patients by one professional” (P2).*

#### Trained staff turnover

Participants had also notified trained staff turnover (within and out of the facility) and replacement of untrained professional at the clinic to cause interruption of the service as described by program coordinators: “*We have a problem in staff turn-over, it is a big problem. There is staff change from facility to facility. There is also within the health facility shifts/changes” (P17). Another participant also described: “When there is trained staff turnover and untrained staffs get assigned in that TB clinic, unknowingly there will be an interruption of the service” (P16).* Participants suggested that problems related to staff turnover could be addressed by organizing opportunities to hand over skills to newly assigned providers. The following quote captured such suggestion: *“If TB focal person who leaves the facility or changes his work unit fails to handover very clearly for the next coming staff, the TB program will fail” (P17).*

#### Sudden withdrawal of partners

Because the programs are highly dependent on partners’ support, the continuity and sustainability of the supply are often affected, and the services fail when the partners’ project ends. This idea was described by a program coordinator as: *“… still we don’t have guarantee to obtain nutrition support … So far this NGO was mainly providing support. But the support is interrupted after the project phase out” (P17)*. A care provider further explained this idea as:

*“… The nutrition program is dependent on partners’ support, so no one gives attention when their support terminates. Because of this, it has no continuity” (P10).*

#### Weak linkage with social service

The weak linkage between sub-city social affairs and health facilities to provide social support for patients with low socioeconomic status was noted to be a barrier. This idea was described by the TB program coordinator as: *“It is very challenging; you go to social affairs office then you might have another step which might be challenging” (P17).* Strengthening communication between the organizations was suggested to solve the problem related to the weak linkage between sub-city social affairs and health facilities.

### Organizational level facilitators of NACS implementation

#### Training

The participants identified that receiving integrated TB nutrition training builds the capacity of health providers and facilitates the implementation of nutrition assessment and counseling. They also reported that it has increased their commitment to giving the service. A care provider stated this as: “*… as we are already educated and trained, we are committed to working …*” *(P7).* A health center director also strengthened this idea in his explanation as: *“Based on the knowledge they have got from the training; they are providing education for the patients” (P12).* In addition, special nutrition training given for some professionals (TB focal persons working on MDR TB clinic) facilitated the implementation of nutrition service and reporting of nutrition-specific issues. A TB program coordinator explained: *“There are professionals specially trained for nutrition service implementation and they also report nutrition-specific things. So, this helps its implementation” (P16).*

In contrast, lack of training for care providers, health facility directors, and case team leaders was noted to hinder the care providers’ engagement and identification of the nutritional problems of the patients. As described by the care provider: *“… we didn’t receive any training about this issue but here is a new registration book which is given to us by the regional health bureau this year. So that, there is nutritional information in the registration book but since I have no idea and full information about NACS, I couldn’t fill it” (P6).* A health center director further noted: *“It is also the lack of training hindering the implementation. If the focal persons are trained, they will be highly engaged and so that they can easily identify gaps. Therefore, they should have a depth of knowledge on the nutrition for the TB patients” (P13).*

Lack of orientation and training for the facility directors and case team leaders was reported to hinder their ability to monitor and supervise the nutritional service given for TB patients that in turn hinders the service implementation. This was described by a care provider: *“The leaders do not understand this service hence no one monitors and follow-up the service and support us” (P10).*

#### Availability of measurement and education tools

The presence of measurement tools and teaching aids in the TB clinic was reported to facilitate the implementation of nutritional assessment and counseling services. A care provider described this as: *“We have a document that guides us to provide nutritional counseling which was given three years back. It has a list of food items like chicken, meat, millets, and others. When we use this document for the provision of counseling, every patient appreciates the counseling” (P8).* This was further described by program coordinators as follows: “… *If there is no measurement scale in the TB clinic then the professionals take the patients to other service outlets to take the anthropometric measurements. This will make them bored, so that they may not do it. But the presence of these measurement tools in their room helps them to do the nutritional assessment. Each of the TB clinics has its weight scale. This facilitates assessment” (P14)*. However, using an un**-**calibrated weight scale hindered proper implementation of nutritional assessment as described by a patient: *“Take this weight balance as an example, look on it, it is just biased, when they weigh us, they say that add 5 or 2 Kg to the reading to get your actual weight. So, there is a barrier related with the instruments and this should be adjusted” (P1).*

#### Inclusion of nutrition indicators in the TB register

The presence of nutrition parameters in the TB register was reported to help supervisors to monitor the implementation of nutrition service as this increases the accountability of care providers to do the nutritional assessment. This idea was described by the health center director as: *“The presence of parameters of nutrition assessment in the TB register helps us to check whether the parameters are fully recorded or not. We also ask why if it is not complete. There is no reason not to be recorded completely. So, the presence of register that comprised the nutrition assessment parameters is helpful” (P13)*. A program coordinator supported this idea as follows: *“The HMIS TB register has all important parameters including nutritional status. So, according to the register, we check all the records. If there are incomplete records, we ask why they missed it” (P14)*. A care provider also described this as: *“… as it is required in the record. Then, if I miss it, it will harm me because there will be a question related to the service provided …” (P8).*

#### Collaborative partners

The follow-up and support of partners were also reported to encourage care providers to work with accountability and facilitate the implementation of the service. A care provider described this as: *“Even we are not working previously; we work due to their presence to supervise us. So, we work to avoid mistakes in the service provision. So far, they check whether the weight and height and the support given are appropriate” (P10)*. He further explained: *“They support us even when there is a shortage of plumpy nut, and they call to Federal Ministry of Health to inform the presence of SAM cases and necessary supports to be given. So, if there are missed services, we will correct them accordingly. Therefore, the follow-up and support of challenge TB have facilitated the implementation of nutrition assessment” (P10).*

### Care providers’ level barrier of NACS implementation

#### Lack of commitment

The participants reported NACS implementation was also influenced by the commitment of the care providers. This was described by a care provider as: *“… due to carelessness of the health professionals, the service is not well done sometimes … Even if you know, you might forget due to lack of attention. I can say, it is due to our weakness, we didn’t give value for it” (P10)*. In addition, subjective judgment of the professionals was also reported to hinder the professional’s commitment to conduct a nutritional assessment for TB patients. A health center director pointed out this idea as: *“… the nurses might not assess if they feel the patient is well-nourished” (P13).*

### Patients’ level barriers of NACS implementation

#### Socioeconomic status of patients

Patients’ socioeconomic status was reported to make the care providers uncomfortable to counsel patients about the nutritional issue because the patients tell the care providers that they cannot afford even to eat twice a day or have no one to support them. This was described by a care provider as: *“Because most of the time when we advise TB patients to eat properly with high protein foods they say “from where can I get those all supplies? It is beyond my economic level. I can’t afford those foods”. So, you know in this case, as a human when you hear this you feel discomfort. Henceforth, it is difficult to say a word about nutrition issues because I couldn’t help them regarding diet” (P6).*

The patients’ socioeconomic status was also reported to affect their compliance with nutritional counseling. A care provider pointed out this as: *“When we counsel to feed properly, our patients ask us “how they can feed, who will support?” they say as they have no one who supports them, so this is a challenge.” (P9)*. This was also described by an MDR-TB patient: *“The implementation mainly depends on economic status. Ideally, I accept the counseling and recommendations from the care providers, however, in practice there are uncertainties; like economic constraints” (P1)*. Another TB patient also described this: *“It depends on the individual/patient whether to apply or not. The patients may decide themselves based on their economic status” (P3).*

In addition, some patients expect nutrition support because their low socioeconomic status was reported to hinder their compliance with nutrition counseling. This was illustrated as: *“… when you tell the patients that their weight has decreased and advised them that they should take food, they expect you to give them nutritional support. They don’t take your advice” (P16).*

#### Sharing and selling of the supply

Participants reported that some patients sell the nutritional supply given to them and buy foods that are nutritionally poor as compared to the plumpy nut or want to use the money for another purpose. A care provider explained: *“… when you give them plumpy nut they decrease its nutritional value by selling it and they want to buy juice” (P10).* In addition, sharing of supply with family was also raised as a barrier to implement the nutritional support properly. The program coordinator described: *“They [patients] may give it for their families, and they may also sell it with the need for money” (P16).*

#### Perceived improved status

Patients’ perception of their prognosis after symptomatic relief from the disease was reported to be a barrier for the implementation of nutrition counseling as the patients perceive that they are okay, so they don’t need the service. This was explained by the TB patient as: *“The patient is eager to receive the counseling because he wants to be free of the pain and coughing which is common during the first month. However, after a bit of follow-up and treatment, during the second month, the patients get relief from cough and pain. Hence, most of the patients perceive that they are fully treated and cured so that they refuse the nutritional counseling. Most of the patients would not follow the counseling after the second month due to their wrong perception” (P2).*

#### Perceived stigma

Perceived stigma –patients who do not want to be considered as TB patients by other people – was reported to be a barrier as this makes them not stay and receive services correctly. This idea was mentioned by a TB patient as*: “I think TB thought of as a shameful disease. Some patients intend to hide and don’t want to be considered TB patients. Some want to come to pick the drug and go back without stay and interact with anyone. So, for the sake of not being seen by others, many patients miss the counseling” (P2).*

### Patients’ level facilitator of NACS implementation

#### Motivation to know their status

Participants reported that motivated patients –those who want to know their status and request to be assessed – motivate their care providers to give them the services. The care provider illustrated: *“The patients request to be assessed and supported nutritionally” (P10). The patient also described: “Some patients are interested to get counseling services. The professionals are interested to deliver the counseling when they meet such patients.” (P2)*. A program coordinator also described this as: *“… there are patients that implement what the health professionals tell them. This motivates the professional’s intention to bring a change in their community” (P14).* In contrast, resistant and hard-to-follow patients were noted to hinder the implementation of the service. This idea was illustrated by the TB program coordinator: *“There are also patients who are very hard to follow like they don’t take the drug, don’t come to the facility even with the call” (P14).*

## Discussion

The current study explored barriers and facilitators of NACS service from organizational, care providers/health professionals, and patients’ perspectives. The emerged themes were linked with the socio-ecologic model that depicts multilevel factors –organizational, care providers, and individual patients –to the implementation of NACS as used elsewhere [28, 31]. Suboptimal nutritional supply, lack of supportive supervision, lack of adequate workforce, staff turn-over, the sudden withdrawal of partners, and weak link with social service were identified as the barriers to the implementation of NACS at the organization level. Lack of commitment was the only barrier at the care provider level and socioeconomic status of patients, sharing and selling of supply, perceived improved status, and perceived stigma were the barriers to the implementation of NACS at the patient level. Similarly, training, availability of measurement and educational tools, the inclusion of nutrition indicators in the TB register, and collaborating partners were identified as facilitators to the implementation of NACS at the organization level, and patient motivation to know their health status was found to be the facilitator at the patient level.

Trained staff, various resources, and administrative supports are often required for a health program implementation [32]. In the present study, suboptimal nutritional supply (lack and shortage) in TB care was reported to be a major barrier that hinders the implementation of NACS. This is in line with a qualitative study from Uganda showing provision of nutrition supply is the most important source of motivation that enables the providers to improve the quality of nutrition services offered [26].

In the current study, lack of supportive supervision was also identified as a barrier to NACS implementation. This is consistent with the assessment report from four regions of Ethiopia –Dire Dawa, Addis Ababa, Oromia, and Amhara – that showed significant improvement in the quality of NACS services for HIV patients due to clinical mentorship [25].

Lack of adequate workforces to provide the service when there is high patient flow and with additional workload was noted to hinder the implementation of NACS. Previous qualitative studies also showed the workload and limited time as barriers to the implementation of nutrition services [31, 33]. In addition, the assessment done in developing countries on monitoring of NACS found shortage of workforce as a barrier for integration of NACS in the existing health system [34]. Moreover, in the current study, trained staff turnover was also pointed out as a barrier to NACS implementation. This finding is consistent with a qualitative study conducted in Northwest Ethiopia that reported as highly trained staff turn-over hampered the quality of TB service [35].

Nutrition service for TB patients was reported to be heavily dependent on the collaborating partners. When the partner program ends, attention and accountability by health care workers and directors become low. In line with this finding, a qualitative study done on NACS implementation for HIV patients in Ethiopia showed that the nutrition services had traditionally been supported by partners but not the regional health bureaus which resulted in low attention and accountability of health staff [25].

Lack of integration of social affairs office and the health facilities to provide support for eligible patients was also found as a barrier that hinders the implementation of nutrition support for TB patients. In line with this, a study from developing countries reported limited ability to link health facilities to community-based economic strengthening and livelihood programs hinders effective implementation of NACS [34]. A qualitative study finding from Peru also showed the importance of social support for the effective implementation of TB programs [36]. Besides, stakeholders’ support has long been found to enhance program implementation as well as sustainability [32].

At the care providers’ level, lack of provider’s commitment was reported as a barrier to the implementation of NACS. This is consistent with findings from a qualitative study that reports professionals’ commitment and motivation as the main facilitator of nutrition service [33].

Poor socioeconomic status of the patient was noted to make care providers uncomfortable to counsel them about the nutritional issue. It was also noted to prevent the patients to comply with the counseling provided for them. This is in line with study findings from Ethiopia that reports economic and food constraints as barriers of compliance to the general TB service that also affects their compliance to nutrition counseling and education [37, 38]. It is also in line with recommendation that shows interventions that address economic and food needs of the entire household are essential to ensure successful treatment of malnourished patients [39].

Sharing and selling of therapeutic food were found to hinder the implementation of NACS in this study. This finding is consistent with a study conducted in Ethiopia that demonstrates sharing and selling of therapeutic food as the main challenge for adherence to therapeutic food among HIV patients [40]. Another qualitative study conducted in Ethiopia indicates caregivers of under-five children perceived therapeutic food as food to be shared and when necessary a commodity to be sold for collective benefits for the household [39].

Perceived wellness after symptomatic relief from the pain by the patients was found to hinder the implementation of NACS. In line with this, a quantitative study shows patients who have the wrong perception about TB disease have higher odds of poor compliance to treatment than their counterparts [41]. It is also consistent with a qualitative study finding from Ethiopia that reports patients have the intention of withdrawing medication due to perceived wellness [42]. Moreover, perceived stigma was found to hinder the implementation of NACS. This finding is consistent with a qualitative study done in Ethiopia that shows stigma as the main problem for TB treatment compliance [43].

Lack of trained professionals and presence of a limited number of TB focal persons were barriers to NACS implementation which is in line with a survey conducted in developing countries on understanding monitoring of NACS [34]. This is also supported by a qualitative study that assessed the challenges in tuberculosis control done in Ethiopia that indicated the absence of training as one of the challenges of TB care [35].

Provision of integrated TB/nutrition training for TB focal persons and health workers emerged as a facilitator of NACS implementation. In line with this, qualitative studies done in Ethiopia and Uganda show strengthening care providers’ nutrition-related capacity enables them to provide quality nutrition services [25, 26]. Moreover, the provision of training for clinical mentors (supervisors) has increased the mentors’ knowledge and skill to properly supervise service providers to give quality NACS service for people with HIV [25].

The presence of anthropometric measurement tools and teaching aids like broachers and leaflets was noted to facilitate the implementation of NACS. This is in line with the survey report showing the presence of equipment as the main facilitator to implement NACS [34].

The inclusion of nutrition parameters in the TB register was identified as a facilitator of NACS implementation in the study. In line with this, an interventional study done in Zambia to improve NACS implementation shows that the introduction of a data recording tool that includes NACS parameters improves its implementation [27]. This could be explained by the fact that routine health data recording encourages the monitoring and evaluation of the overall health system [44].

Moreover, at the organization level, partners that provide either technical or financial support were identified as a facilitator of implementation of NACS. This is in line with international recommendations set as a guide for national TB programs that strongly suggest collaboration and improving coordination with partners for effective implementation of TB programs [45, 46].

Patients’ motivation to be assessed and supported nutritionally was found to be a facilitator that promotes the NACS implementation. This is in line with a qualitative study done in Ethiopia that indicates HIV patients are very motivated to take therapeutic food [47].

Our study has several strengths. The interview guide used was developed through a detailed literature review and reviewed by qualitative research experts that allowed for obtaining in-depth information. The credibility and validity of findings were ensured by continued interviews until data saturation occurred. The research team conducted daily debriefing sessions during data collection to follow emerging issues in the subsequent interviews that further ensured the validity of the study findings. The depth and validity of the findings were also further strengthened by the inclusion of participants from different levels (from the health facility level including patients to the regional level experts). The depth of information was also further ensured through the inclusion of TB patients with different disease statuses (TB/HIV, MDR-TB, and DS-TB) and the place where they are receiving care (hospital and health center).

However, it has also some limitations. To further strengthen the depth of information, the use of multiple data collection methods would have been good. In this regard, the current study used only one method of data collection (key informant interview), thus further research may strengthen the current findings by collecting data through mixed methods. In addition, though interviews were carefully conducted, there could be over-reporting or underreporting of barriers and facilitators of the implementation due to social desirability bias. The similarity of contextual factors in the settings is necessary for the transferability of qualitative evidence [48]. Therefore, the procedure, setting, and context described in this study should be considered to apply the results to other settings. Otherwise, the results generated in this study are largely the descriptions of the context of health facilities in Mekelle City (Mekelle, Ethiopia) concerning barriers and facilitators of NACS implementation.

## Conclusion

Our study is the first to explore the barriers and facilitators of NACS implementation for TB patients. The findings contribute to know the existing strengths and challenges of NACS implementation. Subsequently, the results can be utilized to improve NACS service for TB patients, which in turn helps to improve the nutritional status and quality of life of the patients. More importantly, to achieve the national and global END TB goal by 2035, the health systems should consider the multilevel factors to properly implement the NACS.

The barriers and facilitators of NACS were found to exist at different levels (organization, care providers’, and patients’ level). Therefore, multifaceted approaches are needed for the health system to successfully implement NACS and to gain its potential impact for the TB patients as well as the society. Collaboration between institutions at the health care system, organizations like social affairs, and other relevant stakeholders particularly those supporting the program is also very crucial. Sustained provision of nutrition supply, regular supportive supervision, task sharing, the inclusion of tuberculosis patients in social safety nets and income-generating activities and strengthening nutrition-related capacity and clinical mentorship aimed at boosting the motivation of the care providers are recommended.

## Supplementary Information

**Additional file 1.** Interview guide.

**Additional file 2.** COREQ 32-item checklist for interviews.

## Data Availability

Data based on which conclusions were made are included in this published article.

## Abbreviations

DS-TB: Drug Susceptibile Tuberculosis
HIV: Human Immunodeficiency Virus
NACS: Nutrition Assessment, Couseling and Support
MDR: Multidrug Resistant
MDR-TB: Multidrug Resistant-Tuberculosis
TB: Tuberculosis
TB/HIV: Tuberculosis and Human Immunodeficiency Virus
