# Supplementary material for: Barriers and facilitators of nutrition assessment, counseling, and support for tuberculosis patients: a qualitative study
Source: BMC Nutr. 2021 Oct 13;7:58. doi: 10.1186/s40795-021-00463-x (PMC8513346; doi:10.1186/s40795-021-00463-x)
Supplement: Supplementary file 1 — Additional file 1. Interview guide. [file 40795_2021_463_MOESM1_ESM.docx]

# Interview guide

## Key informant interview guide for program coordinators/ experts/facility managers/TB care providers (focal persons)

**Interviewee details**

1. Woreda(district): ____________________________
2. Name of key informant: ______________________
3. Institution of key informant: ___________________
4. Interviewer name: ___________________________
5. Date of interview: ___________________________
6. Interview start time: _________________________
7. Interview end time: __________________________

**Interviewee professional information**

1. Gender
   1. Female
   2. Male
2. Age: _________ years
3. Highest level of completed education.
   1. College education
   2. Bachelor’s degree
   3. Master’s degree or specialty
4. Profession
   1. Nurse
   2. Health officer
   3. Medicine
   4. Other______________________(specify)
5. Current job/position: ____________________________________________
6. How long have you been in the current job/position?
   1. ______ Months
   2. ______ Years

| S.no | Questions |
| --- | --- |
| 1 | **Routine TB Service** |
| 1.1 | Would you tell me the services that TB clients receive in this health facility?  Probe:   - Would you tell me nutrition related services provided for TB clients? - What services do you think are important for these clients? |
| 1.2 | Would you tell me what you know about nutrition assessment, counseling and support (NACS) service for TB clients?   - Probe for any source of information (training, colleagues/co-workers, media/reading) - What is your opinion on the need to integrate nutrition services with routine TB services? |
|  | Now I am interested in knowing more about the nutrition related services provided to TB clients in this facility/catchment. |
| 2. | **Nutrition Assessment** |
| 2.1 | Would you tell me the nutritional assessment services provided for TB clients in this health facility?   - In your opinion, what makes it necessary for them? - How do you evaluate the implementation of nutrition assessment as per the standard or the guideline? Why?   - Probe: how consistently is provided? |
| 2.2 | In implementation of NACS, what factors in this health institution facilitate the nutritional assessments for the TB clients?   - Probe: for health facility related factors that enhance the implementation of nutrition assessment? [resources, training, guidelines, others] - What professional related factors in this health facility may facilitate the nutrition assessment? - What factors in the patient side may facilitate the assessment? - What else can facilitate the nutrition assessment service? |
| 2.3 | In implementation of NACS, what barriers in this health institution hinder nutritional assessment for TB clients?  Probe: Ask how the factors mentioned operate in the health facility?   - Ask for lived situations about their barrier mentioned in their health facility - Probe: for HF related factors? [resources, training, guidelines, others] - What professional related factors in this health facility may hinder the assessment? - What factors in the patient side may hinder the assessment? [Knowledge, attitude, compliance…] - What else can hinder the nutrition assessment service? |
| 2.4 | In your opinion how these barriers could be solved?   - For these challenges that you mentioned, can you tell me any successes or innovations that your facility/any responsible body has used to improve nutrition assessment implementation? |
| 3. | **Counseling Service** |
| 3.1 | Would you tell me the nutritional counseling services provided for TB clients in this health facility? In your opinion, what makes it necessary for them?   - In the implementation of NACS, how do you evaluate the implementation of counseling for TB patients? |
| 3.2 | In implementation of NACS, what factors in this health institution facilitate the nutritional counseling for the TB clients?   - Probe: for health facility related factors? [resources, training, guidelines, others] - What professional related factors in this health facility may facilitate the nutrition counseling? - What factors in the patient side may facilitate the nutritional counseling? - What else can facilitate the nutrition counseling service? |
| 3.3 | In implementation of NACS, what barriers in this health institution hinder nutritional counseling for TB clients?  Probe: Ask how the factors mentioned operate in the health facility?   - Ask for lived situations about their barrier mentioned in their health facility - Probe: for health facility related factors? [resources, training, guidelines, others] - What professional related factors in this health facility may facilitate the counseling? - What factors in the patient side may facilitate the counseling? [Knowledge, attitude, compliance…] - What else can hinder the nutrition counseling service? |
| 3.4 | In your opinion how these barriers could be solved?   - For these challenges that you mentioned, can you tell me any successes or innovations that your facility/any responsible body has used to improve implementation of nutrition counseling? |
| 4 | **Support** |
| 4.1 | Would you tell me the nutritional support provided for TB clients in this health facility? In your opinion, what makes it necessary for them?   - In the implementation NACS, how do you evaluate the implementation of nutritional support for TB clients? |
| 4.2 | In implementation of NACS, what factors in this health institution facilitate the nutritional support given for the TB clients?   - Probe: for HF related factors? [resources, training, guidelines, others] - What professional related factors in this health facility may facilitate the nutrition support? - What factors in the patient side may facilitate the nutritional support? - what else can facilitate the nutrition support service? |
| 4.3 | In implementation of NACS, what barriers in this health institution hinder nutritional support for TB clients?  Probe: Ask how the factors mentioned operate in the health facility?   - Ask for lived situations about their barrier mentioned in their health facility - Probe: for health facility related factors? [resources, training, guidelines, others] - What professional related factors in this health facility may hinder the support? - What factors in the patient side may hinder the support? [Knowledge, attitude, compliance…] - What else can hinder the nutrition support service? |
| 4.4 | In your opinion how these barriers could be solved?  For these barriers/challenges that you mentioned, can you tell me of any successes or innovations that your facility/bureau/any responsible body has used to improve nutrition support implementation? |
| 5 | **Winding up questions** |
| 5.1 | Can you tell me some of the successful NACS services that you have implemented in your facility/catchment/region?  How did you manage to accomplish it? |
| 5.2 | Is nutrition service considered as one of the objectives in your TB service program plans, and budgets?   - How do you evaluate monitoring and evaluation of NACS implementation by your supervisors/mentors? |
| 6 | **Additional comment** |
| 6.1 | Do you have anything you want to add particularly on the implementation of NACS? |
|  | Thank you for taking the time to discuss these issues with me today. We have learned a lot from you. As I mentioned at the start of the interview, we will remove all identifying information from the transcript of this conversation. We will make sure that no one can identify you from your comments. If you have any concerns or questions, please feel free to contact me (contact info). Thank you very much for your time! |

## Key informant interview guide for TB clients

**Interviewee details**

1. Woreda(district): _________________________________
2. Name of key informant: ___________________________
3. Institution of key informant: ________________________
4. Interviewer name: ________________________________
5. Date of interview: ________________________________
6. Interview start time: ______________________________
7. Interview end time: _______________________________

**Interviewee professional information**

1. Gender
   1. Female
   2. Male
2. Age: _________ years
3. Religion a. Orthodox b. Muslim c. Protestant d. Other…………(specify)
4. Highest level of completed education.
   1. Illiterate
   2. Primary
   3. Secondary
   4. College education
   5. Bachelor’s degree
   6. Master’s degree/specialty
5. Occupation
   1. Farmer
   2. Daily laborer
   3. Housewife
   4. Self employed
   5. Government
   6. Other______________________(specify)
6. How long have you been in the TB treatment ____________(Weeks)?

| S.no | Questions |
| --- | --- |
| 1 | **Routine TB Service** |
| 1.1 | Would you tell me the TB services you receive in this health facility?  Probe: Would you tell me nutrition related services provided to you in this health facility?   - In your opinion what services are important for you? |
| 1.2 | Would you tell me what you know about NACS service provided for TB clients?  Probe: any source of information (training, colleagues/co-workers, media/reading) |
|  | Now I am interested in knowing more about the nutrition services provided in this facility. |
| 2. | **Nutrition Assessment** |
| 2.1 | Would you tell me the nutritional assessment services provided to you in this health facility?   - What do you think about the nutrition assessment service provided for TB client in this health facility? - In your opinion how do you evaluate the implementation of nutrition assessment? Why?   - Probe: how consistently is provided? |
| 2.2 | In your opinion, what factors in this health institution facilitate the nutritional assessments for the TB clients?  Probe: What professional related factors in this health facility may facilitate the nutrition assessment?   - What factors in the patient side may facilitate the assessment? - What else can facilitate the nutrition assessment service? |
| 2.3 | In your opinion, what barriers in this health institution hinder the implementation of nutritional assessment service provided for TB clients?  Probe: What professional related factors in this health facility may hinder the assessment? Ask for lived situations about the barrier in the patient side may hinder nutrition assessment? [Knowledge, attitude, compliance…]   - What else can hinder the nutrition assessment service? |
| 2.4 | In your opinion how these barriers could be solved?   - For these challenges that you mentioned, can you tell me of any successes or innovations that your facility /any responsible body has used to improve nutritional assessment implementation? |
| 3 | **Counseling Service** |
| 3.1 | Would you tell me the nutritional counseling services provided to you in this health facility?   - What do you think about the nutrition counseling service provided for TB client? - In your opinion how do you evaluate the implementation of nutrition counseling? Why?   Probe: how consistently is provided? Its quality? |
| 3.2 | In your opinion, what factors in this health institution facilitate the nutritional counseling for the TB clients?   - Probe: What professional related factors in this health facility may facilitate the nutrition counseling? - What factors in your side may facilitate the nutritional counseling? - What else can facilitate the nutrition counseling service? |
| 3.3 | In your opinion, what barriers in this health institution hinder nutritional counseling for TB clients?  Probe: can you explain how the factors mentioned operate in the health facility?   - What professional related factors in this health facility may facilitate the counseling? - Probe: for HF related factors? [resources, others] - Would tell me your lived situation about factors that hinder the counseling in your side? [Knowledge, attitude, compliance…] - What else can hinder the nutrition counseling service? |
| 3.4 | In your opinion how these barriers could be solved?  For these barriers/challenges that you mentioned, can you tell me of any successes or innovations that your facility/bureau/any responsible body has used to improve nutrition support implementation? |
| 4 | **Support** |
| 4.1 | Would you tell me the nutritional support provided for TB clients in this health facility? In your opinion, what makes it necessary for them?  In your opinion, how do you evaluate the implementation of nutritional support for TB clients? |
| 4.2 | In your opinion, what factors in this health institution facilitate the nutritional support given for the TB clients?  Probe: What professional related factors in this HF may facilitate the nutrition support   - What do you think can facilitate the nutritional support in your side? - What else can facilitate the nutrition support service? |
| 4.3 | In your opinion, what barriers in this health institution hinder nutritional support for TB clients?  Probe: What professional related factors in this HF may hinder the support?   - What factors in your side may hinder the support? - What else can hinder the nutrition support service? |
| 4.4 | In your opinion how these barriers could be solved?  For these barriers/challenges that you mentioned, can you tell me of any successes or innovations that your facility/bureau/any responsible body has used to improve nutrition support implementation? |
| 5 | **Winding up questions** |
| 5.1 | Do you think nutrition services provided for you in this facility are important? |
| 5.2 | How are you benefited from the NACS services provided to you? |
| 6 | **Additional comment** |
| 6.1 | Do you have anything you want to add particularly on the implementation of NACS? |
|  | Thank you for taking the time to discuss these issues with me today. We have learned a lot from you. As I mentioned at the start of the interview, we will remove all identifying information from the transcript of this conversation. We will make sure that no one can identify you from your comments. If you have any questions, please feel free to contact me (contact info). Thank you very much for your time! |
