# Supplementary material for: Barriers and facilitators of nutrition assessment, counseling, and support for tuberculosis patients: a qualitative study
Source: BMC Nutr. 2021 Oct 13;7:58. doi: 10.1186/s40795-021-00463-x (PMC8513346; doi:10.1186/s40795-021-00463-x)
Supplement: Supplementary file 2 — Additional file 2. COREQ 32-item checklist for interviews. [file 40795_2021_463_MOESM2_ESM.docx]

**Additional file 2 – COREQ 32‐item checklist** **for interviews^[[1]](#footnote-1)^**

|  | **Item** |  |  | **Guide questions/description** |  | **Reported on page** |  |
| --- | --- | --- | --- | --- | --- | --- | --- |
| **Domain 1: Research team**  **and reflexivity** | | |  | | |  | |
| *Personal Characteristics* | | |  | | |  | |
| 1. Interviewer/facilitator | | | Which author/s conducted the interview or focus group? | | | Methods, p. 7 | |
| 2. Credentials | | | What were the researcher’s credentials? E.g. PhD, MD | | | Methods, p. 7 | |
| 3. Occupation | | | What was their occupation at the time of the study? | | | Methods, p. 7 | |
| 4. Gender | | | Was the researcher male or female? | | | Methods, p. 7 | |
| 5. Experience and training | | | What experience or training did the researcher have? | | | Methods, p. 7 | |
| *Relationship with*  *participants* | | |  | | |  | |
| 6. Relationship established | | | Was a relationship established prior to study commencement? | | | Methods, p. 7 | |
| 7. Participant knowledge of the interviewer | | | What did the participants know about the researcher? e.g.  personal goals, reasons for doing the research | | | Ethics approval and consent to participate, p.27 | |
| 8. Interviewer  characteristics | | | What characteristics were reported about the   interviewer/facilitator? e.g. Bias, assumptions, reasons and interests in the research topic | | | Methods, p. 8 | |
| **Domain 2: study design** | | |  | | |  | |
| *Theoretical framework* | | |  | | |  | |
| 9. Methodological  orientation and Theory | | | What methodological orientation was stated to underpin the study? e.g. grounded theory, discourse analysis,  ethnography, phenomenology, content analysis | | | Methods, p.6 | |
| *Participant selection* | | |  | | |  | |
| 10. Sampling | | | How were participants selected? e.g. purposive, convenience,  consecutive, snowball | | | Methods, p.6 | |
| 11. Method of approach | | | How were participants approached? e.g. face‐to‐face,   telephone, mail, email | | | Methods, p.7 | |
| 12. Sample size | | | How many participants were in the study? | | | Methods, p.7 | |
| 13. Non‐participation | | | How many people refused to participate or dropped out?  Reasons? | | | Methods, p.8 | |
| *Setting* | | |  | | |  | |
| 14. Setting of data collection | | | Where was the data collected? e.g. home, clinic, workplace | | | Methods, p.8 | |
| 15. Presence of non‐  participants | | | Was anyone else present besides the participants and researchers? | | | Methods, p.7-8 | |
| 16. Description of sample | | | What are the important characteristics of the sample? e.g.   demographic data, date | | | Methods, p.7  Results, p.9 | |
| *Data collection* | | |  | | |  | |
| 17. Interview guide | | | Were questions, prompts, guides provided by the authors?  Was it pilot tested? | | | Methods, p.7-8 | |
| 18. Repeat interviews | | | Were repeat interviews carried out? If yes, how many? | | | Methods, p.7-8 | |
| 19. Audio/visual recording | | | Did the research use audio or visual recording to collect the  data? | | | Methods, p.8 | |
| 20. Field notes | | | Were field notes made during and/or after the interview or   focus group? | | | Methods, p.8 | |

| 21. Duration | What was the duration of the interviews or focus group? | Methods, p.8 |
| --- | --- | --- |
| 22. Data saturation | Was data saturation discussed? | Methods, p.8 |
| 23. Transcripts returned | Were transcripts returned to participants for comment  and/or correction? | Methods, p.8-9 |
| **Domain 3: analysis and findings** |  |  |
| *Data analysis* |  |  |
| 24. Number of data coders | How many data coders coded the data? | Methods, p.9 |
| 25. Description of the  coding tree | Did authors provide a description of the coding tree? | Results, p.10 |
| 26. Derivation of themes | Were themes identified in advance or derived from the data? | Methods, p.9 |
| 27. Software | What software, if applicable, was used to manage the data? | Methods, p.8-9 |
| 28. Participant checking | Did participants provide feedback on the findings? | Methods, p.8-9 |
| *Reporting* |  |  |
| 29. Quotations presented | Were participant quotations presented to illustrate the   themes/findings? Was each quotation identified? e.g.   participant number | Results, p.11-21 |
| 30. Data and findings consistent | Was there consistency between the data presented and the findings? | Results, p.11-21 |
| 31. Clarity of major themes | Were major themes clearly presented in the findings? | Results, p.11-21 |
| 32. Clarity of minor themes | Is there a description of diverse cases or discussion of minor themes? | Results, p.11-21 |

1. Tong A, Sainsbury P, Craig J. Consolidated criteria for reporting qualitative research (COREQ): a 32‐item

   checklist for interviews and focus groups. Int J Qual Health Care. 2007;19(6):349–57. [↑](#footnote-ref-1)
